# Action Perception in Athletes: Expertise Facilitates Perceptual Discrimination

**DOI:** 10.1177/00315125231182046

**Published:** 2023-06-05

**Authors:** Róisín E. Harrison, Martin Giesel, Constanze Hesse

**Affiliations:** 1 School of Psychology, 1019 University of Aberdeen, Aberdeen, UK

**Keywords:** sport, transfer, motor expertise, perceptual resonance, cue, sprint

## Abstract

Prior research has demonstrated that athletes outperform non-athletes on action perception tasks involving anticipation of sport-related actions. We conducted two experiments to determine whether this advantage persists on tasks without anticipation and/or transfers to non-sport actions. In Experiment 1, motor experts (sprinters) and non-experts were shown two consecutive videos of an athlete either walking or sprinting. The participants’ task was to indicate whether the videos were identical or different. The sprinters were more accurate in these judgments than non-experts, indicating that their athleticism was associated with motor expertise that enhanced their perception of both expert and everyday actions. Further analysis revealed that participants who reported basing their decisions on a specific and informative cue (i.e., the distance between where the athlete’s foot landed and a line on the track) outperformed those who did not. However, the sprinters benefitted more from using this cue than the non-sprinters. In Experiment 2, we assessed whether non-experts’ performance improved if the number of available cues was reduced to make the informative cue easier to identify. Non-experts completed the same task as in Experiment 1, with half of the participants viewing the upper part of the athletes’ body and the other half viewing the lower part containing the informative cue. However, the non-experts still did not reliably identify the cue, and performance did not vary between the two non-expert sub-groups. The results of these experiments suggest that motor expertise indirectly affects action perception by improving experts’ ability to identify and use informative cues.

## Introduction

“Watch and learn” is a simple phrase that captures something about how we like to teach and be taught. To learn how to perform a new motor skill, it is helpful to watch someone else perform it before we attempt it ourselves. We seem to inherently acknowledge a relationship between seeing and doing (i.e., between visual perception and action) that is central to several theories of action perception claiming a strong link between visual perception and action (Jeannerod, 2003; Kilner et al., 2007; Wilson & Knoblich, 2005). In fact, these theories propose that we understand the actions of others in relation to our own action capabilities. Presumably, we map the observed action onto our own motor representation of that action during action observation. One prominent theory suggests that perception and action recruit shared representational resources (Prinz, 1997), and that either perceiving or performing a certain action enhances this action’s representation, benefitting both processes going forward. Schütz-Bosbach and Prinz (2007) proposed that the relationship between perception and action is bidirectional; perception can influence action (“motor resonance”), and action can influence perception (“perceptual resonance”). Perceptual resonance was described as an observer’s selective sensitivity to actions that are related to and share features with their own actions. Therefore, perceptual resonance predicts that people who perform certain movements exceptionally well (i.e., motor experts) can also be expected to perceive these movements exceptionally well. Ericsson et al. (1993) described an expert as an individual who has accrued at least ten years or 10,000 hours of deliberate, high-level practice. However, this criterion has not been universally adopted in all motor expertise studies (see Swann et al., 2015 for a review of the inconsistent use of the terms “elite” and “expert” for describing athlete participant samples in sport psychology research). The demographic information provided about the samples used in individual studies is important to contextualize findings along the expert continuum, but we will use the term “motor expert” generally to refer to individuals who regularly train and perform specific and complex motor skills competently.

There is evidence from past studies with motor experts (e.g., athletes and dancers) to support this perceptual resonance prediction, but most of these studies focused on action anticipation. For example, athletes consistently performed better than novices on perceptual tasks in which they needed to anticipate upcoming actions and action effects related to their sport (see Brenton & Müller, 2018; Müller & Abernethy, 2012 for reviews). This superior performance ranged from predicting the success of free-throws (Aglioti et al., 2008) and detecting deception from movement kinematics (Sebanz & Shiffrar, 2009) in basketball to anticipating the direction of badminton strokes (Abernethy & Zawi, 2007). However, it is important to note that perception is not limited to anticipation and can be assessed in many ways. A range of perceptual paradigms should be used to explore perceptual resonance if we are to gain a thorough understanding of the relationship between motor expertise and action perception. Mann et al. (2007) conducted a meta-analysis of studies examining perceptual-cognitive skills in athletes and found that athletes generally outperformed non-athletes on a range of tasks, such as decision-making, anticipation and spatial occlusion. The anticipation research paradigm evoked the largest performance difference on response time and accuracy between athletes and non-athletes, while recognition and recall research paradigms evoked the smallest performance differences. Importantly, however, very few studies employing simple perceptual paradigms, such as detection and discrimination, were included in Mann et al.’s (2007) meta-analysis. Mann et al. (2007) argued that simple tasks may not differentiate athletes and non-athletes, and that this differentiation only occurs with more complex tasks. This may explain the lack of studies examining athletes’ perception of expert actions in simple, non-anticipatory research paradigms involving recognition, detection, and discrimination.

Cañal-Bruland and Williams (2010) observed that different critical movement features were used for recognition tasks than for anticipation tasks, indicating that these processes are quite different. Participants were presented with pairs of dynamic stimuli depicting a leftward or rightward tennis serve that ended at racket-ball contact. On two separate tasks, participants were asked to indicate either the direction of the ensuing shot (anticipation task) or whether the two displays were identical or different (same-or-different/recognition task). The kinematics of the legs, hips, shoulders, trunk, or the arms and racket were manipulated in various conditions to determine the critical features used for recognition and anticipation. The results showed that accuracy was affected differently in the anticipation and recognition tasks, respectively, depending on which body parts were manipulated in the display. Thus, our understanding of motor experts’ visual perception should not rely solely on findings from anticipation tasks.

Most motor expert studies that used simple perceptual tasks used point light displays (PLDs) as stimuli (Calvo-Merino et al., 2010; Cañal-Bruland & Williams, 2010; Hohmann et al., 2011; Romeas & Faubert, 2015). PLDs are animations in which the human body is represented by moving dots that correspond to the body’s major joints, allowing the isolation of kinematic information (Johansson, 1973). For example, Calvo-Merino et al. (2010) reported that professional dancers performed better than novices on a same-or-different task in which participants were shown PLDs of stereotypical ballet moves. The isolation of kinematic information – without the influence of factors such as body shape, identity, and clothing – is a major advantage of PLDs. However, their use as stimuli in motor expertise research may be problematic because they lack ecological validity. Mann et al. (2007) noted that the type of stimulus affected athletes’ performance on perceptual-cognitive tasks and that the likelihood of observing a motor expert advantage was higher when stimuli and research environments were ecologically valid. This may help to explain the generally poor level of discrimination performance exhibited by both groups (i.e., experts and novices) in the Calvo-Merino et al. (2010) study, since participants were likely to have been guessing throughout the experiment. Although task difficulty in motor expertise research prevents a ceiling effect in which both groups do so well that there is no differentiation between groups, task difficulty that results in chance performance from both groups is also problematic. Troje (2013) suggested that PLDs are most appropriate when the researcher’s aim is to investigate the structural reconstruction process that integrates the moving dots into a coherent percept of a moving body. For tasks that require the identification or discrimination of features of the articulated body, this reconstruction process might be an unnecessary and irrelevant complication. In those instances, the use of more ecologically valid stimuli, such as videos, may be more appropriate – especially when the expert advantage is likely to be small.

While evidence of superior perceptual performance from motor experts for expert actions is limited, there has been even less research investigating the transfer of a motor expert advantage to other familiar actions that are unrelated to the domain of expertise. Quarona et al. (2020) discussed both the “specific advantage hypothesis” – whereby experts would be expected to outperform non-experts only for expert actions– and the “general advantage hypothesis” – whereby experts would be expected to outperform non-experts on *both* expert actions and on other actions unrelated to the domain of expertise. Romeas and Faubert (2015) explored this nuanced transfer question when they used an immersive, virtual environment to present soccer athletes and novices with PLDs depicting walking (everyday action) and a soccer kick (expert action). Participants were asked to identify the direction of the walker or predict the trajectory of the masked ball for soccer kick trials. They showed that the athletes generally responded more quickly and accurately on both the soccer kick and the walking trials than did the novices. The athletes’ superior performance on the walking trials may suggest that experts’ enhanced action perception generalized to non-sporting contexts, consistent with the “general advantage hypothesis.” Note, however, that the tasks differed between the two actions; the soccer task involved anticipation, whereas the walking task was a basic direction discrimination task. Therefore, it is difficult to directly compare the performances between the two actions.

In the current experiment, we aimed to determine whether an expert advantage could be observed in an ecologically valid task that did not have an anticipatory component. Our second aim was to assess whether the potential advantage to motor experts was specific to expert actions or whether it generalized to other familiar (everyday) actions. A group of motor experts (track sprinters) and a group of non-experts completed a basic perceptual task in which they were shown videos of an expert action (sprint start) and an everyday action (walking). Sprinters were recruited because they do not interact with other athletes in the same way that team sports players do (e.g., basketball and soccer players, examined previously by Hohmann et al., 2011 and Romeas & Faubert, 2015), but they still exhibit motor expertise that may enhance action perception (Schütz-Bosbach & Prinz, 2007). Therefore, examining sprinters provides an insight into whether perceptual resonance can be observed in athletes for whom observation of other athletes (teammates and competitors) is less crucial to successful sporting performance. Participants were shown two consecutive videos depicting the same action type (i.e., sprinting or walking) in each trial, and were asked to determine whether the videos were identical or different (i.e., a same-or-different task, as in Calvo-Merino et al., 2010 and Cañal-Bruland & Williams, 2010). In trials where the videos were identical, the same video was shown twice. In trials where the videos were different, two separate videos were shown, each depicting the same athlete performing the same action type, but on different occasions. The task aimed to assess participants’ ability to notice subtle kinematic differences between similar/repeated executions of expert actions and everyday actions. The video stimuli provided a more ecologically valid alternative to the PLDs used in previous studies while still controlling for the confounding features of individual bodies (e.g., body shape, identity, clothing). As the pairs of videos always showed the same person performing the same action in the same environment, only the kinematic information differed between videos. The inclusion of a comparison stimulus depicting an everyday action with which motor experts and non-experts should be equally familiar (walking) had two major benefits. First, the use of an everyday action provided a “control” condition in which all participants would be expected to perform well, allowing researchers to assess whether the task was appropriate to answer the research question, independent of motor expertise. If participants struggled with the task in conditions using stimuli depicting everyday actions, the task might be too difficult. Second, this use of an everyday action made it possible to ascertain whether any expert advantage observed was specific to the expert action or generalized more widely. The use of a basic discrimination task addressed the gap in prior research regarding motor expert studies with simple paradigms, and the task parity in the walk and sprint conditions facilitated direct comparisons of these different motor actions (c.f. Romeas & Faubert, 2015). The specific advantage hypothesis predicted that the sprinters would exhibit higher accuracy than the non-sprinters only in the sprint condition (in line with the perceptual resonance hypothesis). The general advantage hypothesis predicted that the sprinters would outperform the non-sprinters in both the sprint and walk conditions.

## Experiment 1: Method

### Participants

We recruited two groups of volunteers for Experiment 1: a motor expert group and a non-expert group. All participants reported that they had normal or corrected-to-normal vision and were naïve to the purpose of the experiment. All participants provided written informed consent before the start of the experiment. The study protocol was approved by the School of Psychology Ethics Committee at University of Aberdeen (PEC/4865/2021/11). Participants were reimbursed with course credit, a Love2Shop voucher, or £12 (∼ $15).

The expert group consisted of 20 track sprinters who regularly trained for athletics and practised sprint starts (10 females, 10 males; *M* age = 22.5 years, *SD* = 3.6 years*;* range: 19–32 years). Participants in the expert group had been involved in athletics for an average of 9.7 years (*SD* = 5 years, range = 0.5 – 19 years) and trained for an average of 9.9 hours (*SD* = 3.1 hours, range = 1 – 12 hours) per week at the time of participation. The non-expert group first consisted of 23 people who did not participate in athletics. Two participants with experience in track and field athletics (but not as a sprinter) were excluded from analysis. While their experience in athletics was not specific to that of a sprinter, they were deemed to still have a type of relevant motor expertise. One other participant had a physical disability and was excluded from analysis for exceptional motor capabilities relative to the rest of the sample. This left a total of 20 participants (10 females, 10 males) in the non-expert group (*M* age = 24.9 years, *SD* = 8.3 years; range: 17–47 years). The athlete sample in the current study is similar in size to other studies in the field: 24 experts in Calvo-Merino et al., (2010); 16 experts in Güldenpenning et al. (2013); 18 experts in Hohmann et al. (2011); 9 experts in Nakamoto et al. (2015); 12 experts in Experiment 1, 14 in Experiment 2 in Sebanz & Shiffrar (2009); 11 experts in Weast et al. (2011).

We used McKay and colleagues’ (2021) classification framework to characterize participants’ level of fitness and performance. This framework was designed to help standardize the terminology used to describe samples of elite athletes in sport science research (see Swann et al., 2015 for a discussion on the importance of standardizing terms in sport psychology research). The framework comprised six tiers: Tier 0 (Sedentary); Tier 1 (Recreationally Active); Tier 2 (Trained/Developmental); Tier 3 (Highly Trained/National Level); Tier 4 (Elite/International) and Tier 5 (World Class). A major advantage of this classification framework was that it could be used to classify the exercise levels of the non-sprinter sample, alongside the performance of the sprinter sample. Tier 0 characterized individuals who did not reach the World Health Organisation’s (WHO) physical activity standards (>150 minutes of moderate activity or >75 minutes of vigorous activity per week). Tier 1 described individuals who met the WHO physical activity standards but did not have a specific commitment to or focus on competition within a particular sport. Tier 2 characterized individuals who participated in sport-specific training and intended to compete in local-level competitions. Athletes did not need to achieve a certain level of performance to be classified into Tier 2. Athletes were, however, required to achieve performance standards to be classified into Tiers 3, 4 and 5. The performance standards used in the current study were adapted from McKay et al. (2021) and calculated from 2021 World Athletics statistics. There were three or four performance indicators associated with each tier, and athletes were put into a certain category if their best performance from the last two years was faster than the mean + the SD of the indicators in each category (see Table A in Supplementary Materials). The expert group comprised nine Tier 2 (Trained/Developmental) sprinters, 10 Tier 3 (Highly Trained/National Level) sprinters and one Tier 4 (Elite/International Level) sprinter. The non-expert group comprised 12 Tier 0 (Sedentary) participants, six Tier 1 (Recreationally Active) participants, one Tier 2 (Trained/Developmental) netball athlete and one Tier 4 (Elite/International Level) kickboxing athlete.

### Apparatus and Stimuli

The experiment was run using a HP Probook Intel® Core i7 laptop and programmed in PsychoPy (Peirce et al., 2019). Stimuli were presented on a 28-inch Iiyama G-master monitor (61 cm × 35 cm, resolution: 2560 × 1440 pixel) with the refresh rate set to 60 Hz. Participants sat at a table in a darkened room at a viewing distance of 68 cm from the monitor. A height-adjustable chin rest was used to maintain a constant viewing distance throughout the experiment. A standard keyboard and mouse were placed on the table in front of the participants. Glow-in-the-dark tape was placed on the keys of interest (“s”, “d” and the spacebar) to highlight the locations of the relevant keys to participants.

The stimuli consisted of short video clips showing a male or female athlete performing a sprint start (expert action) or walking (everyday/control action). Previous researchers have demonstrated a perceptual advantage for watching actions performed by an individual of the observer’s own gender (Bidet-Ildei et al., 2010). Therefore, we used videos of male and female athletes as stimuli and recruited a gender-balanced sample to control for any gender effects. The videos were filmed using a Sony RX100 VII Cyber-shot digital camera set up on a tripod on an indoor athletics track, approximately four meters away from where the athletes were running. After the experimenter provided a verbal “go” signal, the athletes sprinted or walked for approximately eight meters in each video. The athletes were asked to walk and perform sprint starts as they would normally – they were not asked to make adjustments of any kind between repetitions. We filmed 16 videos of each athlete: eight sprint starts and eight walks. Two of the female sprint videos were not used because they were clearly different from the others (e.g., due to stumbling). This left six available videos in the female sprint condition. We then selected the six videos in each of the other conditions (male sprint, female walk, male walk) that were the most similar in duration. The videos for each athlete were filmed in one session and the camera position/lighting conditions did not change during sessions (although conditions may have slightly differed between athletes).

The 24 chosen videos were edited using MATLAB (Version 2018, Mathworks, Natick, MA, USA) to make them more homogenous. The videos were reduced from their original size (1920 × 1080 pixels) to 960 × 540 pixels (approximately 22 cm × 13 cm on the monitor), set to greyscale, and the audio stream was removed. For all videos, the pixel greyscale mean was set to 0.5 and the pixel greyscale SD was set to 0.18. The timestamp of the verbal “go” signal was identified by analysing the audio stream of each video. The sprint videos were trimmed so that they began approximately 150 ms before the “go” signal. The walk videos were trimmed to begin at movement onset. All videos were trimmed to end as soon as the athlete had completely left the frame. The mean durations of the trimmed videos for each condition can be found in Table B in the Supplementary Materials.

### Procedure

Before the beginning of the experiment, participants received verbal task instructions and were asked demographic questions about their age, gender, and sport participation. Participants were then shown an on-screen demonstration of the task consisting of the task instructions, an example of a “same” trial and an example of a “different” trial.

**Figure 1.**
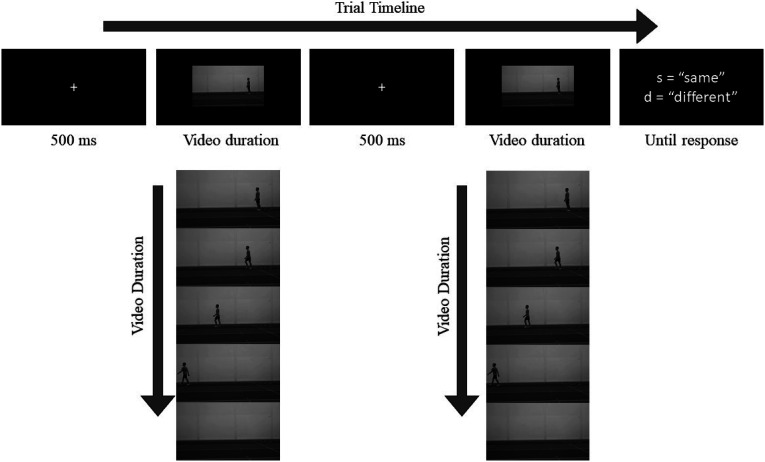
Trial Timeline (Experiment 1).*Note.* The timeline for one trial is shown horizontally. A schematic of a male walk video is shown vertically. Participants pressed the space bar to begin each trial. A fixation cross was shown for 500 ms before the first video was presented in full. Another fixation cross appeared for 500 ms before the second video was played in full. After the second video had finished playing, a response screen appeared with a reminder to “press “s” for same, “d” for different”. The response screen stayed on the screen until the participant responded with either the “s” key or the “d” key.

Figure 1 shows the timeline for each trial. Participants pressed the spacebar to begin each trial. A white fixation cross (1.8 cm × 1.8 cm) appeared in the centre of the screen for 500 ms to signal the start of the trial. Two videos were presented consecutively in the centre of the screen, separated by a 500 ms fixation cross. A response screen appeared after the second video had finished playing. Participants were unable to respond before the onset of the response screen. Once the response screen had appeared, participants were asked to press the “s” key on their keyboard if they thought that the two videos were identical, or the “d” key if they thought the videos were different. In each trial, both videos came from the same condition (i.e., female sprint, female walk, male sprint, or male walk) and trials from the four conditions were presented in a randomized order throughout the experiment. In “same” trials, one video was shown twice. In “different” trials, two separate videos from the same condition were shown, and the differences between the videos reflected natural variations in movement execution. “Same” trials and “different” trials were presented equally often. As there were six videos in each condition, there were 15 unique combinations used for “different” trials. Each combination was shown twice (e.g., video X followed by video Y, and video Y followed by video X), resulting in 30 “different” trials per condition. These “different” trials were complemented with the same number of “same” trials, resulting in 60 trials per condition and a total of 240 trials (4 conditions). Participants could take breaks as often as they liked, and the duration of the experiment was approximately 75 minutes.

### Data Processing and Statistical Analysis

We were primarily interested in participants’ accuracy on the task. We initially calculated participants’ accuracy separately for each of the four conditions (female sprint, female walk, male sprint, and male walk) and ran a two Action (sprint vs. walk) × 2 Depicted Gender (male athlete vs. female athlete) × 2 Participant Gender (male participant vs. female participant) × 2 Expertise (sprinter vs. non-sprinter) mixed factorial analysis of variance (ANOVA) to test for possible gender effects. There was a statistically significant interaction effect between Depicted Gender and Action (*p* = .012) – implying that participants generally performed better on the female sprint videos than the male sprint videos but performed better on the male walk videos than the female walk videos – but, there was no evidence to suggest that participants performed better on the task when the depicted gender matched their own gender (c.f. Bidet-Ildei et al., 2010). As there were no statistically significant main effects of either Depicted Gender (*p* = .452) or Participant Gender (*p* = .474), and no significant interaction effect between these factors (*p* = .361), we combined the data from the male and female videos and across male and female participants to calculate accuracy for the main analysis.

Each participant’s accuracy was calculated separately for the sprint condition (120 trials) and the walk condition (120 trials) and expressed as a proportion correct. These proportion correct scores were then analysed using a 2 Action (sprint vs. walk) × 2 Expertise (sprinter vs. non-sprinter) mixed factorial ANOVA. The data presented here are available online from the Open Science Framework (OSF): https://osf.io/m7d3r/?view_only=3d8372d4a9894f2a8c9a066999300613

### Experiment 1: Results

**Figure 2.**
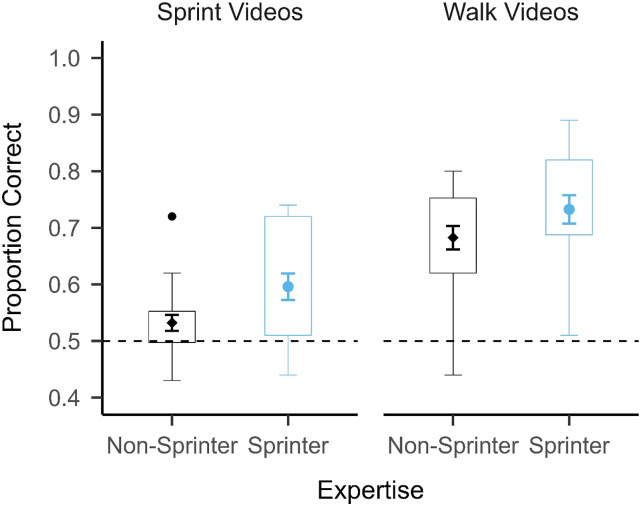
Mean Accuracy and Distribution in Experiment 1 Split by Motor Expertise and Presented Action (N = 40).*Note*. The mean ± 1 standard error of the mean (between participants) is superimposed on a boxplot for each group and action. The bottom of each box in the boxplot represents the 25^th^ percentile for that group, whereas the top of each box represents the 75^th^ percentile for that group. The lower whiskers represent Q1 – 1.5*IQR, whereas the higher whiskers represent Q3 + 1.5*IQR. The horizontal dashed line represents chance performance (0.5). Participants performed better in the walk condition than the sprint condition, and the sprinters outperformed the non-sprinters.

The ANOVA revealed significant main effects of Action: F(1, 38) = 94.349, *p* < .001, η_p_^2^ = 0.713 and Expertise: F(1, 38) = 4.699, *p* = .037, η_p_^2^ = 0.11, but the interaction effect between Action and Expertise was not statistically significant: F(1, 38) = 0.225, *p* = .638, η_p_^2^ = 0.006. These results show that all participants generally performed better on the walk condition (*M* = 0.71, *SD* = 0.11) than the sprint condition (*M* = 0.56, *SD* = 0.09), and that the sprinters (*M* = 0.66, *SD* = 0.13) generally outperformed the non-sprinters (*M* = 0.61, *SD* = 0.11). This can be seen in Figure 2. This data pattern does not support the specific advantage hypothesis; but, rather, it is in line with the general advantage hypothesis, which predicted that the sprinters would perform better than the non-sprinters in both the sprint and walk conditions^1^. We conducted one sample *t*-tests against 0.5 with a Bonferroni adjusted alpha level of .0125 to determine whether the sprinters and non-sprinters generally performed above chance in each condition. The sprinters performed significantly better than chance in both the sprint (*t*(19) = 4.101, *p* < .001, *d* = 0.3) and walk (*t*(19) = 9.217, *p* < .001, *d* = 0.69) conditions, whereas the non-sprinters only performed significantly above chance in the walk condition (*t*(19) = 8.770, *p* < .001, *d* = 0.6) – not the sprint condition (*t*(19) = 2.252, *p* = .036, *d* = 0.12). This suggests that the sprinters were generally able to perform the task for both actions, but the non-sprinters performed at chance in the sprint condition.

**Figure 3.**
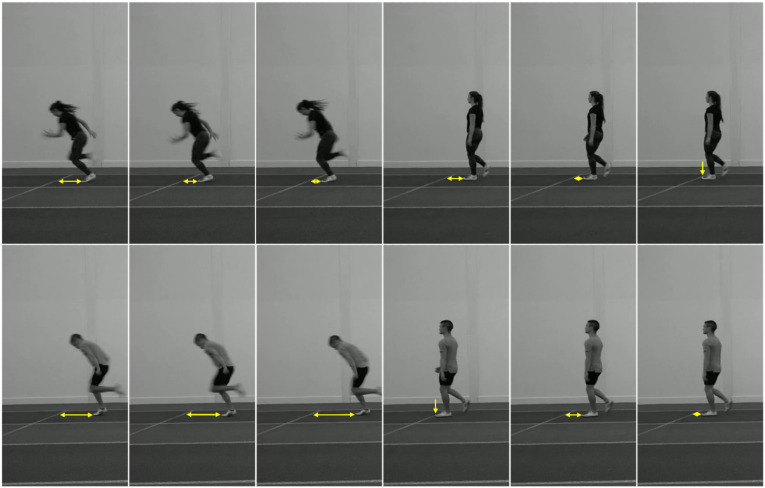
Illustration of the Foot Placement Cue.*Note.* Screenshots taken from 12 of the videos used in Experiment 1. Each image is taken from a different video. The six screenshots on the left are taken from female (top) and male (bottom) sprint videos, whereas the six screenshots on the right are taken from female (top) and male (bottom) walk videos. Each screenshot shows the athletes’ last step before they crossed the line on the track and the yellow arrow represents the distance between where the athlete’s foot landed and the line in each video (i.e., the “foot placement cue”).

During data collection, it became apparent that participants used specific cues to help them complete the task. The experimenter began to ask participants about the strategies they used throughout the experiment. Several participants reported looking at where the athlete’s foot landed in relation to one of the lines on the track and using the distance between the foot and the line (i.e., the “foot placement cue”) to help them make their decision about whether the two videos were the same or different (see Figure 3 for an illustration of the foot placement cue). In an exploratory analysis, we coded the data according to whether the participants reported using this foot placement cue and re-analysed the data. Unfortunately, we did not have information about cue use for all participants because the strategy of cue use only became apparent after the first five participants had been tested. Consequently, re-analyses are based on a total sample of N = 35 participants (18 sprinters and 17 non-sprinters). Seventeen of the 35 participants reported using the cue and 18 did not report using the cue. Descriptively, a larger proportion of the sprinters (12 out of 18) used the foot placement cue than the non-sprinters (five out of 17).

**Figure 4.**
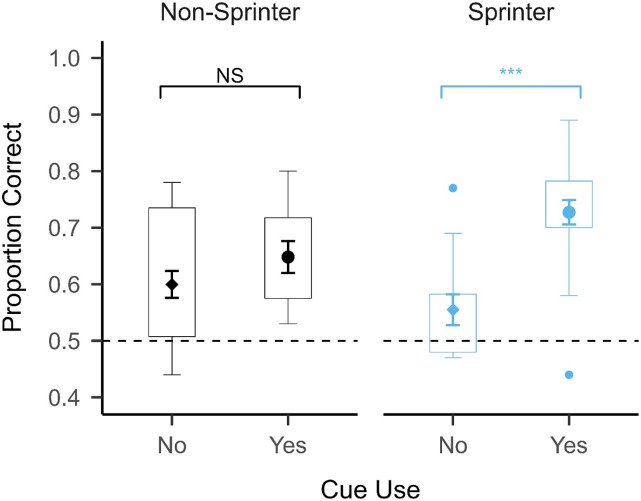
Mean Accuracy and Distribution in Experiment 1 Split by Cue Use and Motor Expertise (N = 35).*Note.* The mean ± 1 standard error of the mean (between participants) is superimposed on a boxplot for each group and separated by whether the foot placement cue was used. The horizontal dashed line represents chance performance (0.5). Sprinters who used the foot placement cue were approximately 0.17 more accurate than sprinters who did not use the cue, whereas non-sprinters who used the cue were only approximately 0.05 more accurate than non-sprinters who did not use the cue.

We analyzed the data using a two Action (sprint vs. walk) × 2 Expertise (sprinter vs. non-sprinter) × 2 Foot Placement Cue Use (used vs. unused) mixed factorial ANOVA. This analysis revealed a significant main effect of Action: F(1, 31) = 51.921, *p* < .001, η_p_^2^ = 0.626 and a significant main effect of Cue Use: F(1, 31) = 23.914, *p* < .001, η_p_^2^ = 0.435. The main effect of Cue Use indicates that participants who used the foot placement cue (*M* = 0.7, *SD* = 0.11) performed better on the task than those who did not use this cue (*M* = 0.58, *SD* = 0.11), which can be seen in Figure 4. The main effect of Expertise was not statistically significant: F(1, 31) = 0.585, *p* = .450, η_p_^2^ = 0.019, unlike in the previous analysis, but the interaction between Expertise and Cue Use was statistically significant: F(1, 31) = 7.522, *p* = .01, η_p_^2^ = .195. Post-hoc, two-sided Welch two-sample *t*-tests revealed that sprinters who used the foot placement cue (*M* = 0.73, *SD* = 0.11) performed significantly better than sprinters who did not use the foot placement cue (*M* = 0.56, *SD* = 0.09): *t*(11.06) = −5.779, *p* < .001, *d* = −2.843. Conversely, there was no significant difference in performance between non-sprinters who used the foot placement cue (*M* = 0.65, *SD* = 0.09) and non-sprinters who did not use the foot placement cue (*M* = 0.6, *SD* = 0.12): *t*(6.367) = −1.311, *p* = 0.235, *d* = - 0.725. This indicates that sprinters generally benefitted more from using the foot placement cue than the non-sprinters. No other interactions were statistically significant (all *p* > .3). These results suggest that the use of the foot placement cue was important for successful task performance, but it is possible that motor expertise and cue use were conflated.

## Experiment 2: Method

The results of Experiment 1 implied that the motor expertise effect was at least partly explained by the use of the foot placement cue, but motor expertise and cue use were difficult to disentangle. The aim of Experiment 2 was to examine whether non-sprinters’ performance could be improved to possibly match the sprinters’ performance in Experiment 1 if the number of available cues was reduced to permit the foot placement cue to be identified more easily during the task. The experimental set-up and procedure of Experiment 2 were very similar to that of Experiment 1, but only non-sprinters were recruited for Experiment 2. Subgroups of this non-sprinter sample underwent altered conditions of only the sprint stimuli. Participants reported using a wider range of informative cues for the walk condition than the sprint condition in Experiment 1 (e.g., walking cadence, leading leg at the beginning of the video), suggesting that the use of the foot placement cue was more important for successful performance in the sprint condition than in the walk condition. Therefore, walk stimuli were not included in Experiment 2. The sprint stimuli from Experiment 1 were edited so that either everything above the knee was hidden (and the foot placement cue was available) or everything below the knee was hidden (and the foot placement cue was unavailable). Participants were assigned to complete the same-or-different task with either the sprint stimuli with the foot placement cue available (“bottom visible” condition) or with the sprint stimuli with the foot placement cue unavailable (“top visible” condition). Participants were given no instructions about where to look or what cues to use. We predicted that participants in the “bottom visible” condition would outperform participants in the “top visible” condition and approach the sprinters’ level of performance in Experiment 1.

### Participants

Thirty-two volunteers with no experience in track sprinting participated in Experiment 2. All participants reported that they had normal or corrected-to-normal vision and were naïve to the purpose of the experiment. All participants provided written informed consent before the start of the experiment and the study was approved by the School of Psychology Ethics Committee at University of Aberdeen (PEC/4947/2022/4). Participants were reimbursed with £8 (∼$10).

Sixteen participants (eight males, seven females, one non-binary; *M* age = 29.9 years, *SD* = 9.1 years, range = 21 – 57 years) were randomly assigned to the “bottom visible” condition (while maintaining gender balance), where the foot placement cue was available. The other 16 participants (eight males, eight females; *M* age = 25.6 years, *SD* = 4.51 years, range = 21 – 38 years) were randomly assigned to the “top visible” condition (while maintaining gender balance), where the foot placement cue was unavailable. Participants were asked to provide details about their exercise habits to allow them to be classified using McKay et al.’s (2021) framework. There were four Tier 0 (Sedentary) participants and 12 Tier 1 (Recreationally Active) participants in each condition.

### Apparatus, Stimuli and Procedure

**Figure 5.**
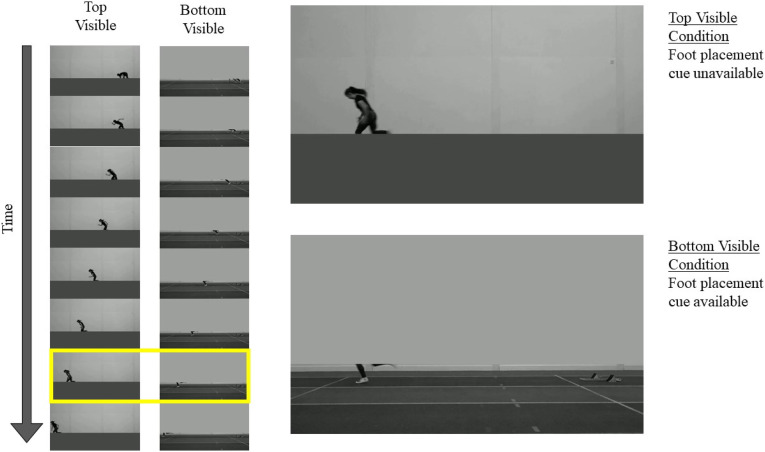
Illustration of the Stimuli Used in Experiment 2. *Note.* Schematics of exemplar videos from the Top Visible and Bottom Visible conditions are represented vertically on the left. The two larger images on the right are taken from the critical point in the videos when the athlete is about to cross the line on the track (highlighted with a yellow box on the left). In the Top Visible condition (top), participants could see most of the athlete’s body, but they could not see where the foot landed in relation to the line on the track (foot placement cue). Conversely, the foot placement cue was available in the Bottom Visible condition (bottom).

The computer and experimental set-up were identical to Experiment 1. Experiment 2 used modified versions of the 12 sprint videos from Experiment 1. A cut-off point along the vertical image axis was determined that was around the height of the athletes’ knees when running in an upright position (depending on the specific phase of the movement, the knees might have been visible above or below this point.). This cut-off point was 325 pixels from the top of the frame for the male athlete and 350 pixels from the top of the frame for the female athlete. Two sets of videos were created from the sprint videos: videos for the “bottom visible” condition and videos for the “top visible” condition. In every frame of the “bottom visible” condition videos, all pixels above the cut-off point were set to the mean pixel value of the pixels above the cut-off point. In every frame of the “top visible” condition videos, all pixels below the cut-off point were set to mean pixel value of the pixels below the cut-off point. This resulted in a homogeneously grey block covering the area either above or below the vertical cut-off point. The lower block was a darker grey than the upper block, consistent with the mean pixel value of the covered area. The mean pixel values and their standard deviations of the resulting frames remained close to those of the videos used in Experiment 1. Figure 5 provides an illustration of the video stimuli used in Experiment 2.

The procedure was identical to Experiment 1. Participants were given the same instructions and were not told what cues to use during the experiment. The duration of Experiment 2 was approximately 40 minutes.

### Data Processing and Statistical Analysis

Each participant’s accuracy in the task was calculated and expressed as proportion correct. An independent samples *t*-test (Welch two-sample *t*-test, two-sided) was applied to the proportion correct scores to determine whether the participants in the “bottom visible” condition were more accurate than the participants in the “top visible” condition.

## Experiment 2: Results

**Figure 6.**
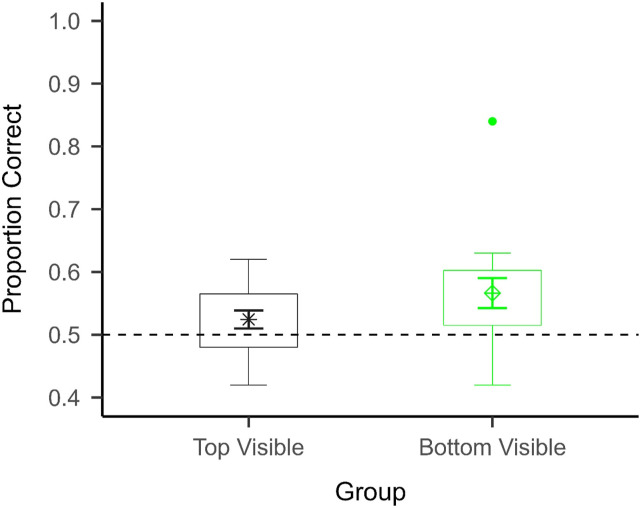
Mean Accuracy and Distribution in Experiment 2 in Split by Condition (N = 32).*Note.* The mean ± 1 standard error of the mean (between participants) is superimposed on a boxplot for each group. The horizontal dashed line represents chance performance (0.5). There was no statistically significant difference in performance between the groups, but only the participants in the “bottom visible” condition performed significantly above chance.

Figure 6 shows participants’ accuracy in Experiment 2. A two-sided Welch two-sample *t*-test revealed that there was no significant difference in performance between participants in the “bottom visible” condition (*M* = 0.57, *SD* = 0.1) and those in the “top visible” condition (*M* = 0.52, *SD* = 0.06): *t*(24.56) = −1.50, *p* = .15, *d* = −0.53. One-sample *t*-tests against 0.5 with a Bonferroni adjusted alpha level of .025 were also run on the accuracy data of each group to determine whether participants generally performed above chance. The one-sample *t*-tests revealed that the participants in the “bottom visible” condition performed significantly better than chance: *t*(15) = 2.77, *p* = .014, *d* = 0.57, but the participants in the “top visible” condition did not perform significantly better than chance: *t*(15) = 1.7, *p* = .11, *d* = 0.1.

Participants in the “bottom visible” condition were not given any instructions to use the foot placement cue. It was expected that they would spontaneously use this cue because there was a lack of other informative cues available in that condition. However, less than half of the participants in the “bottom visible” condition (seven out of 16) reported using the foot placement cue. This suggests that even when the number of cues was significantly reduced, non-sprinters were still less able to identify and use informative cues than sprinters.

## Discussion

The aim of this study was to investigate whether motor expertise enhanced visual perception of expert and/or everyday actions. While previous research has had a strong focus on anticipatory tasks (see Brenton & Müller, 2018; Müller & Abernethy, 2012 for reviews), the aim of the current study was to investigate whether a (generalized or specific) expert advantage could also be observed in a simple perceptual task using ecologically valid stimuli. In Experiment 1, motor experts (track sprinters) and non-experts completed a same-or-different task with videos of an expert action (sprint start) and an everyday action (walking). The specific advantage hypothesis (Quarona et al., 2020) predicted that the sprinters would exhibit higher accuracy than the non-sprinters in the sprint condition only, whereas the general advantage hypothesis predicted that the sprinters would outperform the non-sprinters in both the sprint condition and the walk condition. The sprinters showed better discrimination performance for the sprint and walk videos – consistent with the general advantage hypothesis. An exploratory post-hoc analysis revealed that participants who reported focusing on where the athlete’s foot landed in relation to one of the lines on the track (i.e., used the “foot placement cue”) outperformed participants who did not use this cue throughout the experiment. Furthermore, the sprinters benefitted from using the cue to a greater extent than the non-sprinters, and descriptively a larger proportion of the sprinters spontaneously used this cue than the non-sprinters. Experiment 2 examined whether non-sprinters’ performance could improve if the foot placement cue was easier to identify during the task. One group of participants was shown sprint videos where the foot placement cue was available but everything above the knee was hidden, and another group was shown sprint videos where everything below the knee was hidden, and the foot placement cue was unavailable. We predicted that the group with access to the foot placement cue would outperform the group without access to the foot placement cue. Contrary to our hypothesis, there were no group differences in Experiment 2, but only the group with access to the foot placement cue performed above chance.

Motor experts reliably outperform non-experts on action perception tasks involving anticipation (see Brenton & Müller, 2018; Müller & Abernethy, 2012 for reviews). The few studies that have implemented tasks without an anticipatory component have used point light displays (PLDs) as stimuli, rather than more realistic stimuli used in some anticipation studies (e.g., Aglioti et al., 2008; Cañal-Bruland et al., 2011). The current study addressed these issues by using a perceptual discrimination task with naturalistic video stimuli. Furthermore, we used videos of perceptually similar expert (sprint start) and everyday (walk) actions to assess whether an expert advantage in action perception may transfer to actions outside the experts’ domain of expertise. The results from the primary analysis suggest that the sprinters were better able to notice subtle differences between similar executions of expert actions *and* everyday actions than the non-sprinters. The expert advantage observed in the sprint condition corroborates previous reports that motor experts perform better on basic perceptual tasks involving PLDs of expert actions than non-experts (Calvo-Merino et al., 2010; Hohmann et al., 2011; Romeas & Faubert, 2015). The observation that the sprinters also outperformed the non-sprinters in the walk condition supports the general advantage hypothesis. This suggests that motor experts’ superior ability to perceive expert actions also transfers to other familiar actions outside the domain of expertise (consistent with the findings of Romeas & Faubert, 2015 in soccer athletes).

The transfer of the expert advantage in action perception to similar non-expert actions contradicts the predictions of perceptual resonance (Schütz-Bosbach & Prinz, 2007). Perceptual resonance would predict a specific advantage for the sprinters in the sprint condition (specific advantage hypothesis), not a general advantage across the sprint and walk conditions (general advantage hypothesis) because walking is an action that both groups should be equally motorically familiar with, whereas motoric familiarity with sprinting should be systematically higher in sprinters than non-sprinters. The importance of landmark-related cue use also suggested that perceptual resonance was not the mechanism underlying the sprinters’ advantage on the task (see Philbeck et al., 1997 for an example of using invariant cues to control action). Participants seemed to focus on specific, easily verifiable cues to help them complete the task. Several participants reported that they focused on where the athlete’s foot landed in relation to one of the lines on the track and used the distance between the foot and the line to inform their decision about whether the two videos were the same or different. Overall, participants who used the foot placement cue performed better on the task than participants who did not use this cue – regardless of motor expertise.

**Figure 7.**
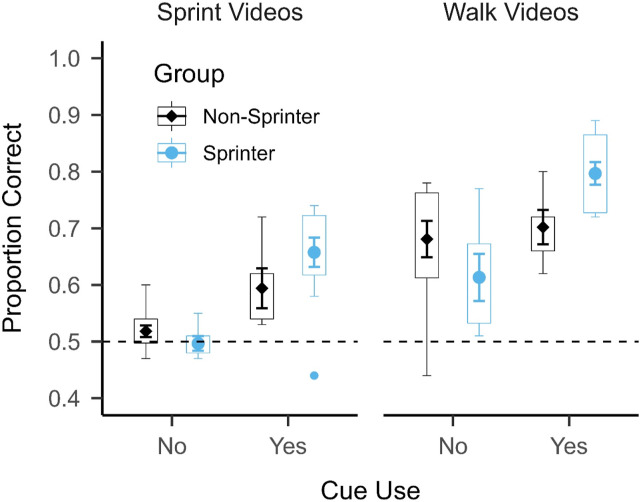
Mean Accuracy and Distribution in Experiment 1 Split by Action, Cue Use and Motor Expertise (N = 35).*Note.* The mean ± 1 standard error of the mean (between participants) is superimposed on a boxplot for each action and group, separated by whether the foot placement cue was used. The horizontal dashed line represents chance performance (0.5). Sprinters who did not use the foot placement cue performed at chance in the sprint condition.

Despite a general advantage for all participants who used the foot placement cue, the sprinters benefitted more from using the cue than the non-sprinters. Sprinters who used the cue performed approximately 17% better than sprinters who did not use the cue, compared to an improvement of only about 5% in the non-sprinters. Most compellingly, sprinters who did not use the foot placement cue performed at chance level (*M* = 0.50, *SD* = 0.03) in the sprint condition (see Figure 7). This suggests that without the cue, sprinters performed similarly to the non-sprinters. These findings do not align with perceptual resonance as it appears that knowledge about where to gather useful information during action observation was more important than the motor experience of performing the action (i.e., motor familiarity/expertise). Vannuscorps and Caramazza (2016) also found that motor experience did not affect action perception as profoundly as has been suggested by theories such as perceptual resonance. Individuals with absent or severely shortened upper limbs (upper limb dysplasia) could perceive upper limb actions as efficiently as typically developed individuals, despite being unable to simulate or perform the actions themselves (Vannuscorps & Caramazza, 2016). This suggested that motor experience (and/or simulation) was not necessary for successful action perception. Despite this, a larger proportion of the sprinters used the foot placement cue than the non-sprinters. This may suggest that motor expertise endows athletes with enhanced knowledge of where to look for useful information during action perception, rather than causing a direct change to the perception of familiar expert actions. In this way, we suggest that the effect of motor expertise on action perception is indirect, which challenges theories that propose a direct, bidirectional link between perception and action (e.g., Schütz-Bosbach & Prinz, 2007).

If knowledge about where to gather useful information during action observation is the key to successful task performance, non-experts who know where to look for useful information should perform better on the task than those who do not. Experiment 2 was devised to test this. One group of non-sprinters was shown versions of the sprint videos where the foot placement cue was available, and the other group was shown versions of the sprint videos where the foot placement cue was unavailable. Contrary to our hypothesis, we observed no group differences in performance. This initially appeared to contradict the idea that cue use could enhance action perception, but further analysis revealed that less than half of the participants in the “bottom visible” condition (seven out of 16) reported using the foot placement cue. This made it difficult to evaluate the importance of the cue in successful task performance because there were not enough instances of the cue being used. Descriptively, participants who used the foot placement cue (*M* = 0.61, *SD* = 0.11) performed better than the other participants with access to the foot placement cue (*M* = 0.53, *SD* = 0.07), and only the group with access to the foot placement cue performed better than chance. While this evidence is insufficient to draw any meaningful conclusions, it does not eliminate cue use as an explanation. At the very least, we can conclude that the non-sprinters did not effectively identify and use the foot placement cue, even when the number of available cues was drastically reduced. This is also in line with the observation that the sprinters benefitted more from using the cue than the non-sprinters in Experiment 1.

Overall, there is reasonable evidence to suggest that experts exhibited enhanced action perception, and that this ability was likely to be underwritten by a higher propensity to identify and use informative cues during action perception. However, one aspect of the study that remains unclear is why a larger proportion of sprinters identified and used the foot placement cue than the non-sprinters. The foot placement cue is not a technical feature of track sprinting that athletes would typically be trained to look for. Important technical features of a sprint start relate to the angle of the forward lean (and the positions of the shoulders, hips, knees, and feet in relation to one another), the amount and direction of force applied, and the speed of movement – particularly in the first few steps of the sprint start.^2^ Conversely, the foot placement cue occurred later in the athletes’ acceleration and had no technical relevance to the sprint start. It is, therefore, surprising that a larger proportion of sprinters spontaneously identified and used this cue throughout the experiment. However, perhaps the sprinters’ advantage was underwritten by a superior ability to identify an element of the sprinting movement that varied sufficiently to discriminate between videos. The important technical features of the sprint start were unlikely to vary drastically between videos because the depicted athletes train to make them consistent between repetitions. In contrast, athletes would not attempt to control the exact landing position of their foot in relation to a line later in the sprinting movement, which may have led to the greater variability of this aspect of the movement between videos. Participants appeared to rely less heavily on the foot placement cue in the walk condition than the sprint condition, presumably because there was a wider range of identifiable, informative cues that varied sufficiently to differentiate videos available in the walk condition (e.g., variation in the leading leg for the first step, visible differences in cadence). The higher accuracy scores in the walk condition than the sprint condition also support this explanation. The sprinters’ perceptual advantage in the sprint condition may have reflected a superior ability to recognize that the technical features of the sprint start were uninformative and to instead focus on an aspect of the movement where there was clearer variability.

### Limitations and Directions for Future Research

The findings from the current study raise questions about expertise, transfer, and cue use for future research to address. Firstly, the observation that the sprinters exhibited an advantage in the walk condition contradicts the view that expertise is situation-specific and that expert-novice differences do not emerge systematically on general tests with non-task-specific stimuli (e.g., Abernethy et al., 1993). However, the study was deliberately designed so that the expert action and the everyday action would be kinematically similar. Walking is perceptually similar to and shares features with sprinting (e.g., both are upright, cyclical actions) and both actions were performed in the same sporting environment (i.e., on an athletics track), which may have made the stimuli more perceptually familiar to the sprinters than the non-sprinters. Therefore, the results suggest that the expert advantage can transfer to another familiar movement unrelated to the domain of expertise, but the extent of this transfer to other everyday actions is still unknown. Further research is needed to better understand the transfer of motor expertise to non-expert domains and determine the limits of task specificity.

These results also suggested that cue use was important for successful task performance, and that it differed between sprinters and non-sprinters. Although the participants’ reliance on the foot placement cue may be considered an artefact and weakness of the stimuli used, the videos were naturalistic, and the strategies used by participants likely reflected how people ordinarily perceive actions. Findings from previous studies with PLDs have shown that motor experts exhibit a perceptual advantage over non-experts when environmental (e.g., lines on the track) and person-specific (e.g., body shape, identity, and clothing) information are not available, but these stimuli may force participants to exercise a different strategy during action perception than they commonly would. A strength of the stimuli used in the current study is that they are ecologically valid. The fact that a portion of the sample relied on a cue that was not directly related to the kinematics of the movements suggests that people may be more comfortable using landmark-related cues rather than trying to assess the movement holistically. Future research could investigate cue use by examining the types of cues that experts tend to discern. One approach could be to vary certain aspects of the movement (e.g., making different information salient and/or easy to verify in different trials) to explore how experts decide which information to use and when. It would also be informative to ascertain whether non-experts can perform as well on an action perception task as experts, if equipped with the knowledge to gather useful information during the movement. Future studies could provide direct instructions to one group of non-experts about how to use a certain cue and compare their performance to a group of non-experts given no instructions about how to perform the task.

## Conclusion

Sprinters were better able to notice subtle differences between similar movement executions of expert actions and everyday actions than non-sprinters. This finding illustrates that expert-novice differences can be observed in basic action perception paradigms and that the expert advantage may transfer to other familiar actions unrelated to the domain of expertise. Participants who focused on an easily verifiable cue during the task tended to perform better than those who did not, but sprinters benefitted more from using the cue than non-sprinters. Despite this, reducing the number of available cues and indirectly encouraging participants to use this cue did not appear to aid non-athletes’ performance in a second experiment. Motor expertise may lead to a superior knowledge of what to look at during a movement and a superior ability to identify and use informative cues.

## Supplemental Material

Supplemental Material - Action Perception in Athletes: Expertise Facilitates Perceptual Discrimination Click here for additional data file. Supplemental Material for Action Perception in Athletes: Expertise Facilitates Perceptual Discrimination by Róisín E. Harrison, Róisín E. Harrison, Constanze Hesse in Perceptual and Motor Skills

## Notes

1. Supplementary data of participants’ hit and false alarm rates are available on the OSF webpage provided on page 11. Most of the analyses of signal detection measures mirrored the accuracy results.

2. Verified by three international-level sprint coaches
