# Supplementary material for: Action Perception in Athletes: Expertise Facilitates Perceptual Discrimination
Source: Percept Mot Skills. 2023 Jun 5;130(4):1472–94. doi: 10.1177/00315125231182046 (PMC10363944; doi:10.1177/00315125231182046)
Supplement: Supplemental Material - Action Perception in Athletes: Expertise Facilitates Perceptual Discrimination [file sj-pdf-1-pms-10.1177_00315125231182046.pdf]

Supplementary Materials

Table A

Performance Standards for Each Tier (seconds).

Gender	Event	World Class	Elite	Highly Trained
Female	60m	7.14	7.53	8.56
Female	60mH	7.9	8.48	9.44
Female	100m	10.74	11.48	12.75
Female	100mH	12.5	13.43	14.81
Female	200m	21.92	23.46	26.04
Female	400m	49.17	53.11	58.25
Female	400mH	52.56	58.59	61.97
Male	60m	6.58	6.91	7.91
Male	60mH	7.44	8.04	8.86
Male	100m	9.92	10.43	11.83
Male	110mH	13.08	13.98	15.45
Male	200m	19.84	20.94	23.74
Male	400m	44.56	46.89	53.31
Male	400mH	46.93	51.06	55.4

Note. The performance standards are expressed in seconds and represent the time that athletes needed to have achieved in the last two years to be classified into a certain tier for a certain event. Each performance standard represents the mean + SD of the performance indicators used for each tier. In accordance with McKay et al. (2021), the performance indicators used for the World Class tier were: i) the world record as of 2021; ii) the 2021 world lead; iii) 2% of the world record; iv) 2% of the 2021 world lead. The performance indicators used for the Elite tier were: i) the 300th ranked performance in the world in 2021; ii) 7% of the world record; iii) 7% of the 2021 world lead. The performance indicators used for the Highly Trained tier were: i) the 2021 entry standard for the British Championships (outdoors, where possible); ii) 20% of the world record; iii) 20% of the world lead.

Table B

Mean Durations of the Trimmed Videos Used in the Experiment

Action	Gender	Mean	SD	Minimum	Maximum	Range
Sprint	Female	2.0	0.02	1.98	2.02	0.04
Sprint	Male	1.87	0.03	1.84	1.9	0.06
Walk	Female	5.16	0.22	4.9	5.4	0.5
Walk	Male	5.06	0.35	4.7	5.74	1.04

Note. The mean durations are expressed in seconds. The mean duration for each condition was calculated from the 6 videos in that condition.

Video Consent

Please cross each box

☒ I understand that video recording equipment is used to film stimuli that will be used in upcoming experiments.

☒ I understand that I can reject the use of my video recordings and they will be deleted immediately without any negative consequences.

☒ I understand that if I give permission, the recordings will be held confidentially so that only the experimenters (Róisín Harrison, Dr Constanze Hesse and Dr Martin Giesel) have access to the video recordings. The recordings will be stored on a password protected server for up to 5 years, after which period they will be deleted. In accordance with the General Data Protection Regulation (GDPR) I can have access to my recording and can request it to be deleted at any time during this period.

☒ I understand that this consent form will be stored electronically on a password protected server.

I allow the video recordings to be used for the following purposes:

1. Scientific research.

☒ YES ☐NO

2. Presentation as an illustration of the above scientific research, in scientific publications.

☒ YES ☐NO

3. Presentation as an illustration of the above scientific research, in professional conferences.

☒ YES ☐NO

4. Presentation as an illustration of the above scientific research, in classes.

☒ YES ☐NO

5. Presentation as an illustration of the above scientific research, on social media.

☒ YES ☐NO

6. Presentation as an illustration of the above scientific research, on online academic homepages.

☒ YES ☐NO

Under no circumstances, will your personal information (e.g., name) be used in the presentation of the recording.

**MHIA MOUAT**	Mhia Mouat	**12/10/2021**
Name (PRINT)	Signed	Date

**RÓISÍN HARRISON**	Róisín Harrison	**12/10/2021**
Experimenter (PRINT)	Signed	Date
